# Reconstruction of a Pelvic Nonunion Secondary to Metastatic Breast Cancer and Palliative Radiation

**DOI:** 10.7759/cureus.90052

**Published:** 2025-08-14

**Authors:** Ronald Peirish, Landon Reading, Cesar Cereijo

**Affiliations:** 1 Orthopedic Surgery, OhioHealth Grant Medical Center, Columbus, USA; 2 Orthopedic Surgery, Cleveland Clinic - South Pointe Hospital, Warrensville Heights, USA

**Keywords:** fragility fracture of the pelvis, insufficiency fractures, nonunion, pelvic reconstruction, radiation

## Abstract

Pathologic pelvic nonunions are uncommon, although they remain a difficult problem and require meticulous surgical planning and execution for desired outcomes. In this paper, we discuss a patient who sustained a pathologic pelvic insufficiency fracture secondary to radiation therapy for metastatic breast cancer, who subsequently developed a symptomatic nonunion that required surgical intervention secondary to debilitating pain and inability to ambulate. We also discuss the classification of insufficiency fractures of the pelvis to guide treatment, in addition to complications associated with surgical intervention. A 51-year-old female with a 12-year history of metastatic breast cancer. She presented to an outside facility where she was diagnosed with a nondisplaced left superior pubic ramus and iliac wing fracture with lytic lesions throughout her pelvis (Rommens fragility fractures of the pelvis (FFP) type IVc). Follow-up at our institution with updated imaging demonstrated a windswept pelvis, left iliac crescent fracture, and bilateral parasymphyseal fracture involving the superior and inferior rami, with a right-sided zone 1 sacral fracture. Discussion with the patient was made regarding conservative nonoperative management, as well as operative management; the patient wished to proceed with surgery, given her debilitating pain and inability to ambulate. The patient was placed into the supine position on a radiolucent table. A urinary catheter was placed. A stoppa approach was performed to access bilateral pelvic brims and the symphysis, along with a lateral window. The left hemi-pelvis was reduced with lateral bookwalter traction. The iliac crest was reduced and stabilized with a 6-hole 3.5 mm recon locking plate and a 7.3 mm fully treated cannulated screw across the LC-2 corridor. A 20-hole 3.5 mm recon plate was used to stabilize the anterior pelvis. Two right-sided 7.3 mm fully threaded cannulated trans-sacral screws with a washer were placed through the S1 and S2 corridors to stabilize the posterior pelvic ring. A mixture of 20cc of demineralized bone matrix (DBX) and allograft was used over the fracture sites. Two months postoperatively, she had increased pain in the right buttocks and had audible clicking with ambulation. Imaging demonstrated a broken S1 transsacral screw and plate anteriorly. The patient then underwent multiple revision surgeries, given hardware failure and progression of her fragility fractures, which are further discussed in the case report. This case is unique as there are no reported cases to our knowledge on the clinical outcome after reconstruction of pelvic nonunion cases secondary to radiation therapy for metastatic breast cancer.

## Introduction

Insufficiency fractures occur when a weakened bone is exposed to a normal load and everyday stressors [1]. High-energy traumas are necessary to cause pelvic injuries in healthy bones; however, pelvic insufficiency fractures can occur in the absence of trauma, from low-energy injuries, or spontaneously [1,2]. Such fractures can lead to malunion and nonunion. A study looking at women with metastatic breast cancer found that around 56% of these women had metastatic disease involving the pelvis [3]. Women undergoing pelvic radiotherapy had a pelvic insufficiency fracture rate ranging from 5 to 20% [4]. Pathologic pelvic nonunions are uncommon, making up 7% of all pelvic nonunion cases in general [5]. Patients most commonly present with new-onset pain in the groin or low back areas but can also present with abnormalities in gait, pelvic instability, and deformity [6]. If left untreated, pelvic nonunion can lead to leg length abnormalities, difficulty ambulating, pain with sitting, and back pain [6].

Several risk factors for developing a nonunion have been identified, including but not limited to chronic steroid use, renal osteodystrophy, diabetes mellitus, alcohol abuse, smoking, and pelvic radiation [1]. Radiation therapy damages osteoblasts and devascularizes the bone, leading to osteopenia [1,7]. Radiation coupled with osteoporosis in elderly patients, particularly postmenopausal women, further increases the risk for insufficiency fractures [7]. Radiation-induced insufficiency fractures tend to affect weight-bearing structures and thus most commonly involve the sacroiliac area [1]. Insufficiency fractures may go unidentified, especially in the setting of radiation therapy, as they may be mistaken for bone metastases [1,7]. Bilateral lesions are more likely to be insufficiency fractures rather than metastases; however, insufficiency fractures can present unilaterally [7]. Pelvic nonunions secondary to radiation have the worst prognosis. This poor prognosis is due to the destruction of the normal anatomy secondary to abundant scar tissue, making surgery increasingly difficult with limited ability of the bone to heal post-operatively [8].

Surgical correction of nonunion pelvic fractures has many known complications. Surgical complications include neurologic injury (5.3%), pulmonary embolism (1.9%), deep infection (1.6%), bladder injuries (0.8%), and vascular injuries (0.5%) [6]. Non-operative treatment can be employed with subsequent healing for most pelvic insufficiency fractures; however, a 2009 study by Kanakaris et al. suggested that nonoperative treatment, in addition to poor initial fracture reduction and sole treatment using external fixation, increased the risk of complications [6]. Matta et al. reported union rates of 83% with nonoperative treatment [9]. Given the complexity of treating these pelvic nonunion cases, patients should be referred to specialized centers.

In this paper, we discuss a patient who sustained a pathologic pelvic insufficiency fracture secondary to radiation therapy for metastatic breast cancer, who subsequently developed a symptomatic nonunion that required surgical intervention secondary to debilitating pain and inability to ambulate. This case report aims to describe the clinical course and surgical reconstruction of a rare pelvic nonunion secondary to radiation for metastatic breast cancer, in addition to complications associated with surgical intervention.

## Case presentation

A 51-year-old female with a 12-year history of metastatic ER/PR+, HER2- breast cancer presented to our clinic for evaluation of a complex nonunion of her pelvis. Her oncologic treatment consisted of a left radical mastectomy, bilateral salpingo-oophorectomy, chemotherapy, and radiation to her thoracic, lumbar, and pelvic regions.

**Figure 1:**
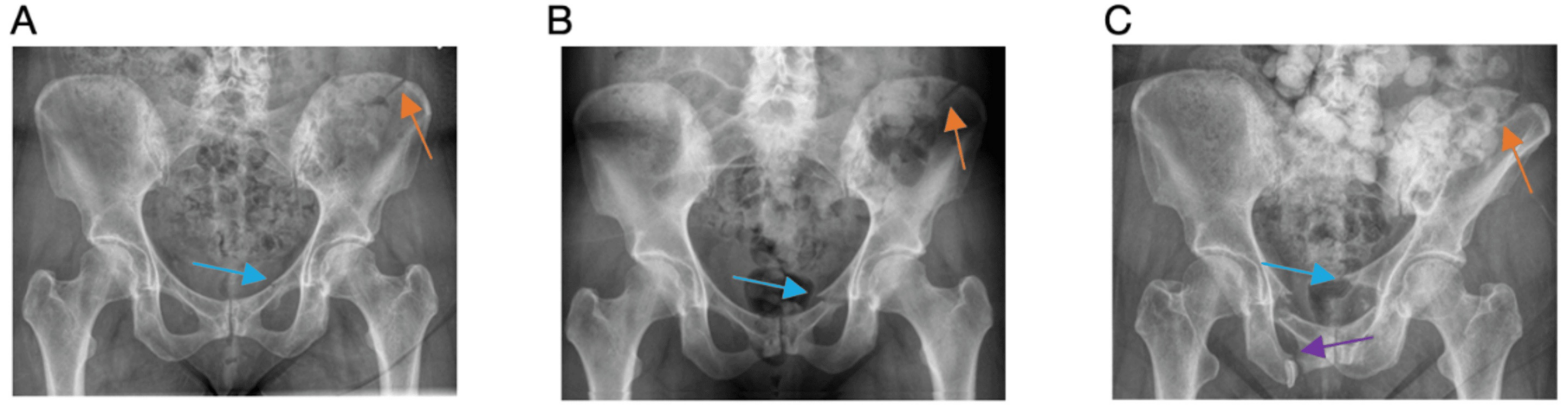
Radiographs demonstrating multiple pathologic pelvic fractures Image A shows a left superior pubic ramus fracture (blue arrow) and left iliac crest fracture (orange arrow). Image B shows more displacement of both the superior pubic ramus fracture (blue arrow) and left iliac crest fracture (orange arrow). Image C shows worsening displacement of both prior fractures of the left superior pubic ramus (blue arrow) and left iliac crest fracture (orange arrow); there is also a new right-sided superior and inferior pubic ramus fracture (purple arrow).

A year and a half prior to presentation, she developed severe atraumatic left-sided coxalgia one month after radiation. She presented to an outside facility, in which radiographs demonstrated a nondisplaced left superior pubic ramus and iliac wing fracture (fragility fractures of the pelvis (FFP) type IIC) with lytic lesions throughout her pelvis (Figure 1A). One week later, she was getting out of the shower and felt a pop in her left hip and was unable to ambulate secondary to pain. Repeat imaging demonstrated displacement in her left superior rami (Figure 1B). Protected weight bearing was initiated.

**Figure 2:**
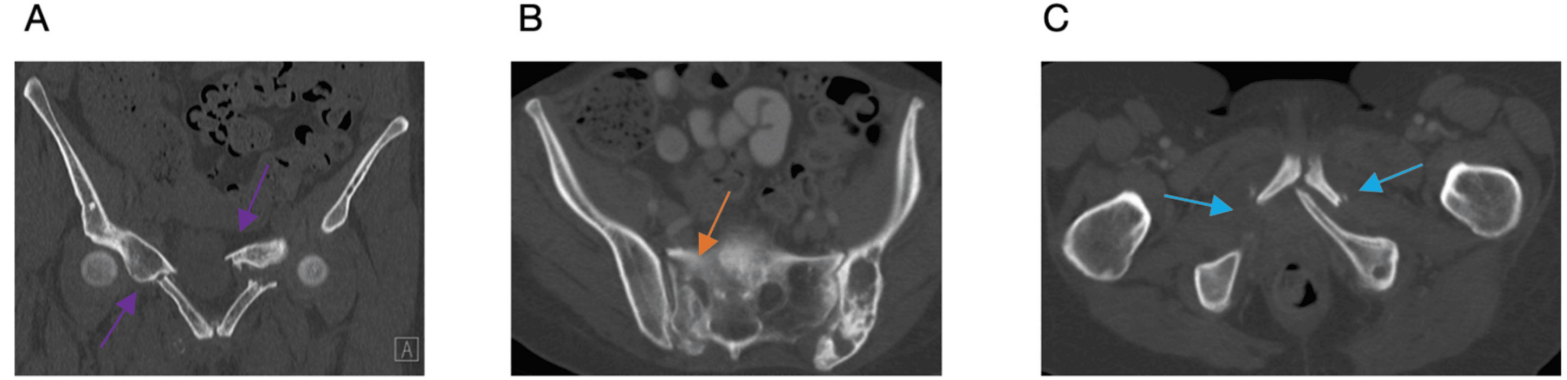
Computed tomography (CT) for surgical planning Image A demonstrates bilateral pubic ramus fractures (purple arrows). Image B demonstrates zone I sacrum fracture (orange arrow). Image C demonstrates bilateral parasymphyseal fractures (blue arrows).

At the time of our evaluation in the clinic, the patient was no longer able to ambulate secondary to debilitating pain over her pubic symphysis and right sacroiliac joint. She was unable to find comfort in sitting or lying down and would awake multiple times throughout the night secondary to pain. Her pain was not controlled with a combination of Tylenol, Advil, gabapentin, oxycodone, and fentanyl. Her pain was reproducible with palpation. Repeat radiographs demonstrated further fracture displacement and additional pelvic insufficiency fractures (Figure 1C). Computed tomography (CT) was obtained, which demonstrated a windswept pelvis, left iliac crescent fracture, bilateral parasymphyseal fracture involving the superior and inferior rami with a right-sided zone 1 sacral fracture (Rommens FFP type IVc). All fracture sites were ununited (Figures 2A-2C).

Nonoperative and operative intervention was discussed with the patient. The primary objective of operative intervention was to provide pain relief and to allow her to sit and lie down in comfort. The goal was to allow her the ability to walk without pain. She elected to proceed with surgery, and a surgical consent was obtained.

**Figure 3:**
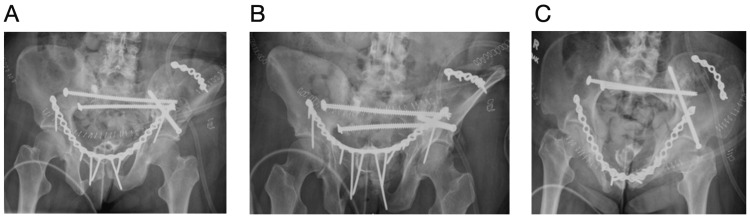
Post op radiographs Images A (AP pelvis) , B (pelvic outlet), and C (pelvic inlet) demonstrate the status post fixation for multiple pathological fragility fractures. The iliac crest fracture was stabilized with a 6-hole plate. A 20-hole plate was used to stabilize the anterior pelvis. There are three cannulated screws, one through the LC-2 corridor, one in the S1 corridor, and one in the S2 corridor.

The patient was brought to the operating room table and placed supine on a radiolucent table. A urinary catheter was placed, and the surgical field was prepared and draped in standard sterile fashion. The Stoppa approach was utilized to identify the anterior aspects of the bilateral hemipelvis. Cystotomy occurred during the intrapelvic approach during manipulation of the rectus secondary to extensive adhesions from her radiation treatment and chronic deformity of the pubic rami, weakening the bladder wall. Urology repaired the bladder wall prior to proceeding. Fracture nonunions were debrided and mobilized. The left hemipelvis was reduced with lateral bookwalter traction. The iliac crest was reduced and stabilized with a 6-hole 3.5 mm recon locking plate, with a 7.3 mm fully threaded cannulated screw across the LC-2 corridor. A 20-hole 3.5 recon plate was used to stabilize the anterior pelvis. Two right-sided 7.3 mm fully threaded cannulated trans-sacral screws with a washer were placed through the S1 and S2 corridors to stabilize the posterior pelvic ring (Figures 3A-3C). A mixture of 20cc of demineralized bone matrix (DBX) and allograft was used over the fracture sites. A hemovac drain was placed in the space of Retzius anterior to the bladder. She was made weight-bearing as tolerated to the right lower extremity and non-weight-bearing to the left lower extremity. Her Foley catheter was removed at two weeks, and a cystogram confirmed no leak. At two weeks post operatively, she reported significant improvement in her pain with a reduction in her baseline narcotics. She was able to sleep throughout the night in comfort. She was able to ambulate through her right lower extremity with a walker.

**Figure 4:**
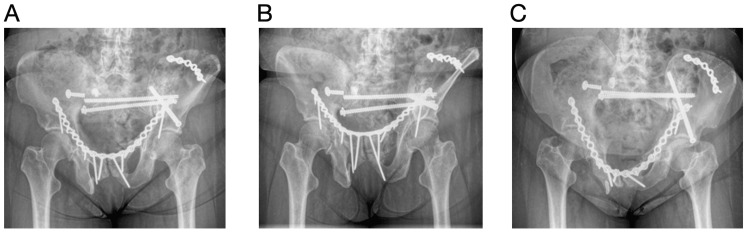
Two month post op radiographs Images A (AP pelvis) , B (pelvic outlet), and C (pelvic inlet) demonstrate the broken S1 transsacral screw and broken plate anteriorly.

**Figure 5:**
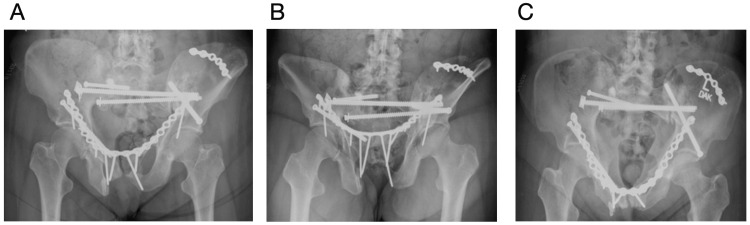
Revision surgery post op radiographs Images A (AP pelvis), B (pelvic outlet), and C (pelvic inlet) demonstrate removal of the previous broken S1 transsacral screw with exchange to a larger cannulated screw in the same S1 transsacral corridor, as well as an additional screw in the S1 iliosacral corridor. An antegrade anterior column cannulated screw was also placed to provide additional support to anterior column.

Two months post operatively, she experienced increasing pain in the right buttocks and had audible clicking with ambulation. Radiographs (Figures 4A-4C) and a CT demonstrated a broken S1 transsacral screw and plate anteriorly. Prior to this, she was ambulating and was able to sit and sleep without discomfort. She was taken back to the operating room, and the broken S1 transsacral screw was removed and replaced with an 8.0 mm fully threaded cannulated screw, and a S1 iliosacral 7.3 mm partially threaded cannulated screw with a washer was placed. The anterior plate was maintained, but an antegrade anterior column 6.5 mm fully threaded cannulated screw was placed to provide additional support to the anterior column (Figures 5A-5C). Post operatively, she was pivot transfers only to the right lower extremity. She was progressed to weight-bearing as tolerated at her two-week follow-up in the office.

**Figure 6:**
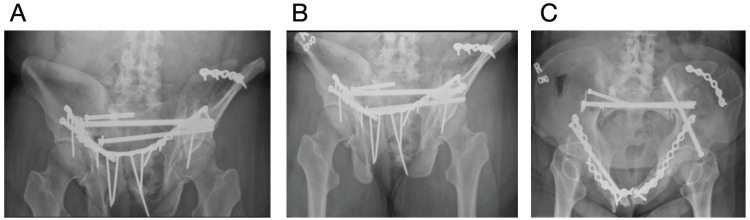
Three-month status post revision surgery radiographs Images A (AP pelvis), B (pelvic outlet), and C (pelvic inlet) show the anterior plate to be broken in two different places with anterior displacement. The images also show a broken left LC-2 screw at the level of the iliac wing.

**Figure 7:**
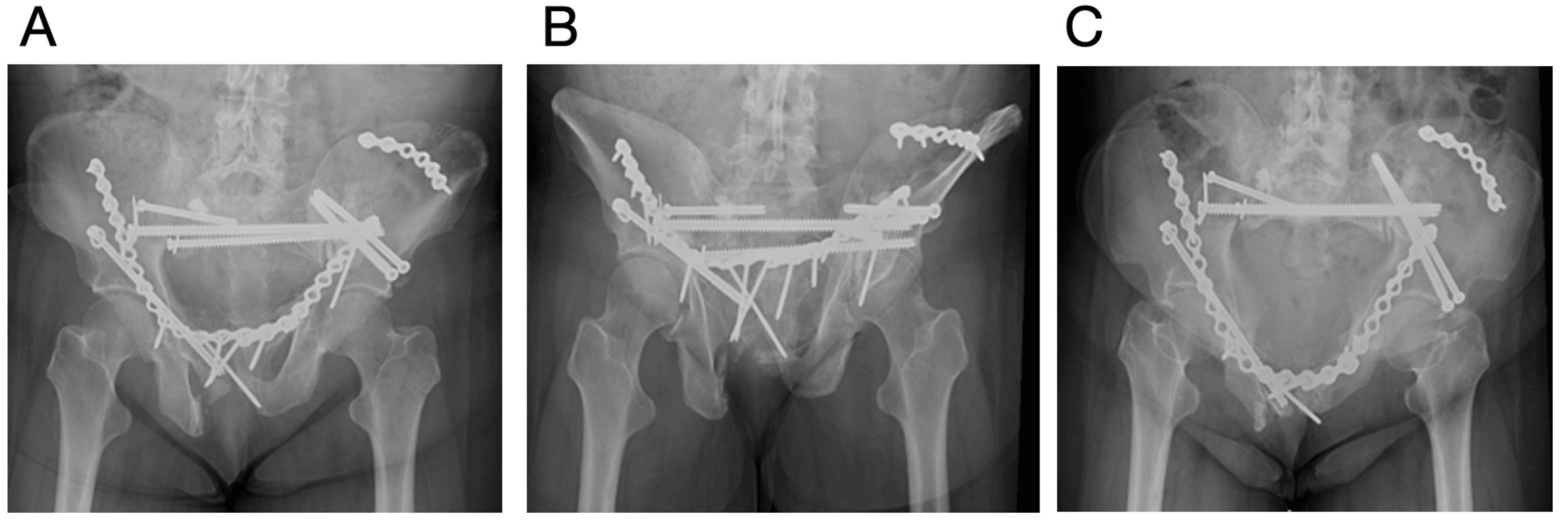
Radiograph status post re-revision surgery Images A (AP pelvis), B (pelvic outlet), and C (pelvic inlet) show the partial removal of the anterior plate with a new 16-hole recon plate overlapping the retained previous anterior plate. The previously placed right anterior column screw was removed and exchanged for a cannulated screw with a washer. The left LC-2 screw was maintained, but an additional left LC-2 screw was placed.

Three months following her revision surgery, she reported new clicking and right groin pain with the inability to ambulate secondary to pain. Radiographs (Figures 6A-6C) and a CT demonstrated her anterior pelvis plate to be broken in two places with anterior displacement and the left LC-2 screw to be broken at the level of the iliac wing. The patient was taken back to surgery for revision. The broken anterior plate was partially removed, and a new 16-hole 3.5 mm recon plate was placed overlapping the retained anterior plate. The previously placed right anterior column screw was removed as it had loosened, and a new 7.3 mm partially threaded cannulated screw with a washer was placed. The left LC-2 screw was maintained, but an additional left LC-2 7.3 mm partially threaded cannulated screw was placed just lateral to the pre-existing screw (Figures 7A-7C). During the intrapelvic approach, a small tear was noted in the bladder, which was repaired by urology at the time of injury. The patient was pivot transfers bilaterally. Her Foley catheter remained until a cystogram at two weeks confirmed no leak. The patient was seen two weeks post operatively and reported significant improvement in her pain. She progressed to weight-bearing as tolerated with the use of a walker two months post operatively. At two months, she reported right sacroiliac pain, but otherwise her pain was minimal. Six months post operatively, she was ambulating with a walker and/or cane. Ten months post operatively, she passed away from the progression of her metastatic cancer.

## Discussion

In 2013, Rommen et al. classified FFP based on the degree of instability to help decide whether initial surgical intervention should be pursued [2]. Four categories were established. FFP type I are isolated anterior pelvic ring fractures, which are treated with conservative measures due to their relative stability [2]. FFP type II are non-displaced posterior ring fractures, which are initially treated with conservative measures, but if this fails, operative intervention is warranted via percutaneous screw fixation [2]. FFP type III are unilaterally displaced posterior pelvic ring fractures, and FFP type IV are bilaterally displaced posterior pelvic ring fractures [2]. Operative intervention is recommended for both FFP types III and IV, with open reduction and internal fixation being the recommended modality for type III and iliolumbar fixation being recommended for type IV [2]. Unfortunately, this classification by Rommen and Hofmann does not provide guidance on nonunion cases. The decision for surgery is based on the amount of pelvic pain, instability, and deformity. Conservative treatment is acceptable when early mobilization can be obtained, but if this is unattainable, then operative intervention should be recommended. Prolonged immobilization increases the risk of decubitus ulcers, deep venous thrombosis, pulmonary embolism, urinary tract infection, delirium, anxiety, and depression.

There is limited literature about the surgical outcomes for pathologic pelvic nonunion. Mears et al. reported that eight out of 44 patients with pelvic nonunion post surgery had persistent pelvic pain at one and two years post operatively [8]. Seven of the patients required additional surgery. Six of the seven patients who had pelvic radiation complained of persistent pelvic pain at one and two years. All these patients had radiographic union within six months [8]. Kanakaris et al. found that surgical correction achieved nonunion healing rates of 86.1%, pain relief in 92.6%, patient satisfaction in 79.4%, and return to preinjury level of activity in 55.9% [6]. They also described common post-surgical complications, with the majority being neurologic injuries and clotting, in addition to organ injury, infection, implant failure, continued nonunion, incomplete reduction, and misalignment [6,10].

Our case is unique in that our patient initially sustained a type IIC FFP, which rapidly progressed to a type IVC FFP. There are limited reported cases to our knowledge that demonstrate this amount of progression from initial injury. In addition, insufficiency fractures of the pelvis secondary to radiation therapy for rectal, anal, cervical, and prostate cancer have been reported in the literature [1,7]. In the future, we would recommend additional strategies or augmented strategies to provide as much fixation as possible, such as hydroxyapatite-coated pelvic fixation screws, larger diameter screws, and additional pharmacological agents to help with bone, such as bisphosphonates. However, this patient had a history of osteonecrosis of the jaw, and so bisphosphonate use was contraindicated. There are no reported cases to our knowledge on the clinical outcome after reconstruction of pelvic nonunion cases secondary to radiation therapy for metastatic breast cancer.

## Conclusions

Pelvic nonunion is a rare sequela following radiation-induced insufficiency fractures of the pelvis. Patients with risk factors for pelvic insufficiency fractures, particularly patients who have undergone or are currently undergoing radiation, should be carefully and promptly evaluated when presenting with new-onset pelvic pain, gait disturbances, and/or signs of pelvic instability or deformity. It is important to distinguish metastases from insufficiency fractures to choose the best treatment plan. Conservative management may be indicated for stable, minimally displaced fractures; however, most will be treated surgically, particularly if there is significant pain, instability, and/or deformity. Classification of fragility fractures of the pelvis, along with clinical judgment, will aid in choosing the most appropriate surgical modality. Surgical intervention is associated with complications, particularly neurologic injury and clotting; however, it is also associated with nonunion healing and pain relief.
